# Editorial: Statistical Relational Artificial Intelligence

**DOI:** 10.3389/frobt.2019.00068

**Published:** 2019-07-30

**Authors:** Fabrizio Riguzzi, Kristian Kersting, Marco Lippi, Sriraam Natarajan

**Affiliations:** ^1^Department of Mathematics and Computer Science, University of Ferrara, Ferrara, Italy; ^2^Computer Science Department and Centre for Cognitive Science, TU Darmstadt, Darmstadt, Germany; ^3^Department of Sciences and Methods for Engineering, University of Modena and Reggio Emilia, Reggio Emilia, Italy; ^4^Erik Jonsson School of Engineering and Computer Science, The University of Texas at Dallas, Richardson, TX, United States

**Keywords:** statistical relational artificial intelligence, statistical relational learning, graphical models, logic in AI, machine learning

## Introduction

Statistical Relational Artificial Intelligence (StarAI) aims at integrating logical (or relational) AI with probabilistic (or statistical) AI (De Raedt et al., 2016; Riguzzi, 2018). Relational AI achieved impressive results in structured machine learning and data mining, especially in bio- and chemo-informatics. Statistical AI is based on probabilistic (graphical) models that enable efficient reasoning and learning, and that have been applied to a wide variety of fields such as diagnosis, network communication, computational biology, computer vision, and robotics. Ultimately, StarAI may provide good starting points for developing *Systems AI*—the computational and mathematical modeling of complex AI systems—and in turn an engineering discipline for Artificial Intelligence and Machine Learning.

This Research Topic “Statistical Relational Artificial Intelligence” aims at presenting an overview of the latest approaches in StarAI. This topic was followed by a summer school^1^held in 2018 in Ferrara, Italy, as part of the series of Advanced Courses on AI (ACAI) promoted by the European Association for Artificial Intelligence.

## Papers Included in This Research Topic

Previous issues on similar topics in other journals and books mainly focused on modeling, learning purely from data and/or lifted inference. This issue went further and addressed more novel and pertinent topics such as grammatical inference, human preferences, and modeling large graphs to name a few.

Vu et al. apply Statistical Relational Learning to the problem of grammatical inference of formal languages. They extend previous works by considering a new set of string position features that relax the requirement of mutual exclusion.

Odom and Natarajan explore the contribution that humans can provide to StarAI systems in terms of extra domain advice. The article shows that human advice can effectively accelerate learning in noisy, structured domains.

Orsini et al. propose a new representation for large graphs that are decomposed in a hierarchical fashion. A feedforward neural network is then unfolded over the hierarchical decomposition. The approach is able to outperform current state-of-the-art graph classification methods on large social network datasets.

Kazemi and Poole investigate the relationship between the path ranking algorithm, a well-known relational learning method based on the random walk paradigm, and relational logistic regression, one of the recent developments in weighted rule learning. The paper shows that relational logistic regression using normalized relations generalizes the path ranking algorithm.

Dragone et al. discuss how preferences can be elicited from human decision makers when the problem is constructive in nature, i.e., when the goal is to synthesize a custom or entirely novel configuration. This setting poses specific problems because the set of possible outcomes is very large.

## Conclusions and Outlook

AI is everywhere, yet major limitations are holding back the realization of its true capabilities. We are currently experiencing the second wave of AI, dominated by deep and statistical learning approaches. But the third wave of AI systems will provide contextual adaptation. They will understand context and meaning, reason about it and be able to adapt accordingly. They will not only recognize a bottleneck in a production line, but will be able to explain what is going wrong and how it arrived at that conclusion. The papers of this Research Topic show that this dream is not insurmountable.

## Author Contributions

All authors listed have made a substantial, direct and intellectual contribution to the work, and approved it for publication.

### Conflict of Interest Statement

The authors declare that the research was conducted in the absence of any commercial or financial relationships that could be construed as a potential conflict of interest.

## Footnotes

^1^http://acai2018.unife.it/
